# MOT: A Low-Latency, Multichannel Wireless Surface Electromyography Acquisition System Based on the AD8232 Front-End

**DOI:** 10.3390/s25123600

**Published:** 2025-06-07

**Authors:** Augusto Tetsuo Prado Inafuco, Pablo Machoski, Daniel Prado Campos, Sergio Francisco Pichorim, José Jair Alves Mendes Junior

**Affiliations:** 1 Graduate Program in Electrical and Computing Engineering, Federal University of Technology, Curitiba 80230-901, PR, Brazil; inafuco@alunos.utfpr.edu (A.T.P.I.); pichorim@utfpr.edu.br (S.F.P.); 2 Department of Electronics, Federal University of Technology, Curitiba 80230-901, PR, Brazil; pablomachoski@alunos.utfpr.edu.br; 3 Graduate Program in Biomedical Engineering, Federal University of Technology, Curitiba 80230-901, PR, Brazil; danielcampos@utfpr.edu.br; 4 Department of Informatics, Federal University of Technology, Apucarana 86812-460, PR, Brazil

**Keywords:** biomedical instrumentation, surface electromyography, AD8232 module, biopotential acquisition

## Abstract

Commercial wearable systems for surface electromyography (sEMG) acquisition often trade bandwidth, synchronization, and battery life for miniaturization, and their proprietary designs inhibit reproducibility and cost-effective customization. To address these limitations, we developed MOT, a fully wireless, multichannel platform built from commodity components that can be replicated in academic laboratories. Each sensor node integrates an AD8232 analog front-end configured for 19–690 Hz bandwidth (59 dB mid-band gain) with a 12-bit successive approximation ADC sampling at 1 kS/s. Packets of 120 samples are broadcast via the low-latency ESP-NOW 2.45 GHz protocol to a central hub, which timestamps and streams data to a host PC over USB-UART. Bench tests confirmed the analog response and showed mains interference at least 40 dB below voluntary contraction levels; the cumulative packet loss remained below 0.5% for six simultaneous channels at 100 m line-of-sight, with end-to-end latency under 3 ms. A 180 mAh Li-ion cell was used to power each node for 1.8 h of continuous operation at 100 mA average draw, and the complete sensor, including enclosure, was found to weigh 22 g. MOT reduced a 60 Hz artifact magnitude by up to 22 dB while preserving signal bandwidth. The hardware, therefore, provides a compact and economical solution for biomechanics, rehabilitation, and human–machine interface research that demands mobile, high-fidelity sEMG acquisition.

## 1. Introduction

The acquisition of surface electromyography (sEMG) involves developing devices able to capture and analyze the signals provided by electrical potentials of muscle contractions [1,2,3,4]. These signals are used in applications, such as monitoring movement patterns, diagnosing medical conditions, ergonomics analysis with exoskeleton, human–machine interfaces, and control of devices, such as prostheses and robotic manipulators [4,5,6,7,8,9,10,11,12,13,14,15]. In addition to that, the use of wearable devices has increased in recent years [16].

In addition, several commercial devices have been developed for the acquisition and capture of the sEMG signals, especially wearable devices, e.g., the Myo™ armband (although discontinued, it is still present in several studies [7,17]) from Thalmic Labs and gForcePro+EMG from Oyomotion [18]. Wearable devices aid the acquisition process, making it more comfortable for individuals during their use and reducing or eliminating the presence of cables. Both devices mentioned above feature eight channels, dry electrodes, and wireless communication for transmitting data collected from upper limbs, which were applied for human–machine interfaces [10,11,12] and gesture recognition [8,9,13,14,19] in general.

Other sEMG devices are utilized for research purposes. Bitalino for Plux Biosignals, Trigo for Delsys, and Miotool from Miotec are some examples of these platforms [20,21,22]. Although these systems were designed to be wearable like Myo and gforcePro, they could offer flexibility for placing acquisition channels in other parts of the human body, not only in the forearm. Some of them, such as Trigno, have no cable in the acquisition. They utilize wireless data transmission to attenuate electromagnetic interference and allow more freedom to data acquisition once the subject is not connected to a physical system by cables and wires.

Although these devices are commercially available, certain limitations, such as technical specifications or cost, motivate researchers to build and make their own sEMG acquisition circuit. One of these limitations is the sampling rate. For instance, Phinyomark et al. demonstrated that lowering the sampling rate from 1 kHz to the 200 Hz used by the Myo armband produces a statistically significant reduction in hand and finger movement classification accuracy, especially for trans-radial amputees, where no combination of conventional features could compensate for the information lost at this lower bandwidth [23]. Examples of devices developed by researchers are the works of [24,25]. In [24], a multichannel sEMG system was developed based on the ADS1293 front-end circuit, ranging from the amplifier to the communication protocol. On the other hand, [25] integrated a board to acquire sEMG (Myoware) with Raspberry Pi Pico, achieving similar precision when compared with other acquisition systems.

Both commercial and research devices have the same intrinsic features in their behavior. An sEMG acquisition circuit has particular steps, which include amplifiers with high impedance; high Common-Mode Rejection Ratio (CMRR) and gain; filters to attenuate noises from movement artifacts; high frequencies; electromagnetic interference (regarding the main power supply); and circuits to condition the signal and allow its conversion for digital format. The main differences are related to the topology of the circuits, the integrated circuits employed, the cutoff frequencies of filters, and the gain used in conditioning steps. These factors play an essential role in hardware development, once the sEMG signal has small amplitudes, in the order of µV until 10 mV and for frequencies in the range of 10–500 Hz, with signals with more energy up to 200 Hz [4,26,27,28,29].

In addition to the features above, some other functionalities are found in acquisition devices for sEMG signals. Among them, one can cite the preference for wireless communication, a system for recording or processing with visualization and storage in real time, low power consumption, and an interface with control devices. Several features are wanted in devices because they aid data acquisition, especially in dynamic conditions and in noise attenuation, such as from movement artifacts and electromagnetic interference [30]. For example, these last two issues could be resolved when cables are not used and wireless communication is employed [31,32,33]. Especially for wireless transmission, such as radio frequency (RF), this directly assists in noise reduction and mobility to users during an acquisition protocol [32,34]. Moreover, decreasing the length of cables helps to place the device near the muscles’ region of interest [30,33,34,35] and, consequently, reduce its weight.

Acquisition device development involves not only the construction of a printed circuit board (PCB) or choosing components but also the software elements, such as firmware and the usability of the interfaces, which favors the adoption of one device over another. Technical factors such as battery life and lightness are also crucial for acquiring sEMG signals in long-duration and high-intensity activities [36,37,38,39]. They allow for a broader range of possibilities for sEMG acquisition devices for various research and application conditions beyond controlled laboratory experiments.

Thus, efficient, accurate, and portable solutions for sEMG signals are crucial. Therefore, this paper presents the development of a wireless sEMG and low-latency acquisition system. It is a multichannel device based on the acquisition circuit AD8232, data transmission based on wireless communication (RF of 2.45 GHz), and data recording for future processing. The platform is called MOT, which is derived from “Motion”, once it allows mobility for the user. The platform includes six sEMG devices with wireless communication, a central receiver, and signal visualization and recording software. The main contribution of this work is to detail the development steps, including sEMG acquisition (hardware), data transmission (firmware), and the interface to device control (software). The main contributions are the following:

- A review of the state-of-the-art methods of sEMG acquisition systems from academic works and commercial solutions is presented to focus on the main features of these devices.
- In this paper, we designed hardware, firmware, and software for multichannel sEMG acquisition.
- We evaluate a full sEMG system development, from electronics design to human–machine interface.

## 2. Related Works

Developing sEMG signal acquisition devices and platforms is a hot topic in biomedical instrumentation, with several devices and approaches being employed in academic development. The analysis was split into two main assessments: devices developed and used by researchers in academic works and available commercial systems.

Researchers’ works were filtered from 2011 to 2023; the main works and the developed system (MOT) the results on Table 1. Systems described in [40,41,42] presented the column “Wearable” that are empty are because the authors include no information about the final device. The only device classified as non-wearable by the authors was the one described by [43], although it had wearable dimensions (67 mm × 45 mm) and was supplied by batteries.

The third column contains information about the data transmission methods, which are (in descending order) Bluetooth (traditional or low consumption—BLE), radio frequency (RF), Wi-Fi, and wired transmission. Except for the wired transmission, the other three approaches work in similar frequency bands—close to 2.4 GHz. In addition, the work indicates that in the RF description, it was not discriminated which method was employed; only the term RF was mentioned, and a frequency band close to 2.4 GHz was employed.

Some works are not only used for sEMG but can be employed for other biopotentials, which is the reason the “Channels” column is marked with ‘-’ [38,40,41,42].

Both the bandwidth and sampling frequency columns directly relate to the quality of the obtained signal. The systems that present the Fs information have an operational maximum frequency close to half an Fs, which is in conformity with the Nyquist criterion. The work of [44] is an exception, as it presents a bandwidth of 15 Hz to 1 kHz. One can note that the most repeated value is 1 kHz for sampling frequency and a frequency band ranging up to 500 Hz.

The last column presents the AD resolution in bits. The most used resolution was 10 bits, followed by 12 and 24 bits. Aiming to reach this resolution, the researchers mainly adopted two approaches: the use of internal ADC from a microcontroller [24,38,45] or the use of external ADC from a converter [43,46]. The use of analog front-ends, as in the ADS1299 device employed by [47], is one alternative way in which a second microcontroller interfaces the data for a control device.

Among the analyzed works, three were chosen to be mentioned due to their architecture and evaluation of the devices. The work of [46] used commercial integrated circuits, such as AD7606 (Analog Devices) for acquisition and nRF52840 for data transmission using the BLE protocol. Data were acquired before the transmission using FIFO (first in, first out) queues. Messages had timestamps for synchronization, and time intervals were calculated between the emitter and receiver. Simultaneous multiple-channel synchronization was not evaluated, and outliers in data transmission were discarded. On the other hand, [38] developed an integrated system with multiple sensors for sEMG acquisition. Each device used amplifiers with Serial Peripheral Interface (SPI) communication with a CC3200 microcontroller. The master device transmitted the data after the recording to a computer using WiFi. However, the authors did not perform any tests to evaluate the data transmission of the system. Similar to the elements in [38], the work of [48] can acquire signals from sEMG and electroencephalogram (EEG) signals using an ADS1299 with a microcontroller to receive and transmit the data to a computer using Wi-Fi. This work highlights their elevated number of simultaneous channels (16 channels) and high resolution of the ADC. The operational bandwidth was not specified, and the authors showed different responses for the gains in different frequencies, observing a band of rejection in 250 Hz due to the ADS1299. However, the authors considered that it did not impact the acquisition once the main frequencies of the muscle signal were below 150 Hz [26].

Following the search for scientific and research works published about sEMG acquisition, Table 1 presents similar devices for muscle signal acquisition. Among the presented systems, the Trigno Centro from the Delsys company [49] stands out due to its sampling frequency and number of channels. According to its documentation, the system can operate in the range of 20 Hz to 450 Hz and 10 to 850 Hz. It uses the BLE communication protocol and can operate with ANT+, but only one protocol can be active during the acquisition [50]. The system operates similarly to the architectures presented in the mentioned works: specialized sensors for sEMG acquisition, a central unit to receive the data, and a final interface where the collected data can be viewed and saved if necessary.

As another example, the commercial DataLITE system, uses dry electrodes connected by double-sided tape to the user, offering 8 h of battery life and a range of 30 m in data communication. In this system, a reference wire must be attached to a place distant from the muscle region of interest. Its sampling frequency is 1 kHz, it has eight channels, and it features 12-bit resolution [51]. On the other hand, the Myo Armband has a sampling frequency of 200 Hz, eight channels, and 8-bit resolution. The FreeEMG system has 10 channels, 16-bit resolution, and a sampling frequency of 1 kHz, with a battery life of up to 6 h and a range of 20 m [52]. This system communication is based on the IEEE 802.15.4 protocol [53]. Due to the protocol’s characteristics, it is impossible to determine whether the system uses Bluetooth, ZigBee, 6lowPAN, or TinyOS—this information is not present in the official documentation.

Except for the FreeEMG system, all other devices utilize the BLE protocol for data transmission. Additionally, the Myo armband and gForcePro+ EMG Armband devices have sampling rates below 1 kHz, and sEMG signals will be captured only for frequencies lower than 100 Hz and 250 Hz, respectively, for each system. Furthermore, none of these systems indicate if data are missing during the wireless transmission. A point to highlight is that the Bitalino system uses the AD8232 IC as an analog interface (with a gain of 1009) to the user.

Due to these factors, the goal of developing the MOT system is to create an sEMG system capable of ensuring channel synchronization, a user-friendly interface to display and save data for posterior processing, and a biopotential hardware acquisition that works within the sEMG’s bandwidth.

**Table 1:** Main features from devices of related researchers published between 2011 and 2023 focused on sEMG acquisition, commercial devices, and the developed system (MOT). BW, Fs, and ADC indicate the frequency bandwidth, sampling frequency, and bit resolution of analog–digital converter, respectively.

Work	Wearable	Data Transmission	Channels	BW (Hz)	Fs (kHz)	ADC (bit)
[44]	Yes	Wired	8	15–1000	1	-
[54]	Yes	RF	6	20–500	1	10
[55]	Yes	Wired	16	20–500	1	-
[43]	No	BLE	4	8.7–952	4	14
[45]	Yes	BLE	2	-	-	12
[38]	Yes	WiFi	-	-	32	24
[47]	Yes	Bluetooth	8	-	0.25–1	24
[40]	-	No	-	20–500	1	16
[39]	Yes	RF	1	-	2	10
[24]	Yes	BLE	3	<633	32	24
[56]	Yes	RF	-	0.07–100	-	10
[42]	-	No	-	10–1000	4	10
[34]	Yes	RF	4	<500	1	16
[41]	-	RF	-	0.65–1000	-	10
[57]	Yes	No	1	25.67–472.9	-	10
[46]	Yes	Bluetooth	8	20–500	1	16
[58]	Yes	WiFi	8	20–500	1	12
[59]	Yes	BLE	1	20–450	1	12
[48]	Yes	WiFi	16	-	1	24
[25]	Yes	WiFi	1	20–450	2	12
DataLITE [51]	No	BLE	8	20–460	1	14
Trigno Centro [49]	Yes	BLE	32	20–450	4.37	16
Myo Armband	Yes	BLE	8	-	0.2	8
FreeEMG [52]	Yes	-	10	-	1	16
Bitalino [60]	Yes	BLE	4	25–482	1	10
gForcePro+ EMG	Yes	BLE	8	-	0.5	12
This work: MOT	Yes	RF (ESP-NOW)	6	19–692	1	12

## 3. Materials and Methods

**Figure 1:**
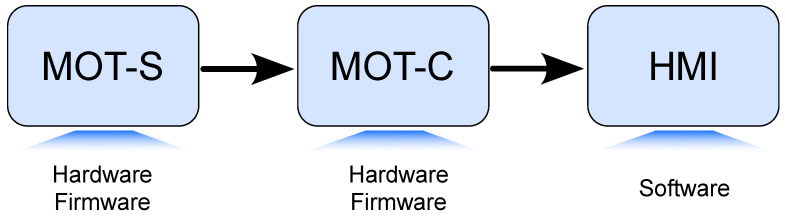
Sequence of MOT development.

This section presents the development of the device, including details of the hardware, firmware, and the experiments conducted to evaluate its performance. The sequence of the MOT development is illustrated in Figure 1. The firmware was developed using Visual Studio Code (ver. 1.86) and the C++ programming language (C++17). EasyEDA Pro (ver. 2.0.32) was employed for the hardware design for PCB and SolidWorks (2023) for the system’s case, while the software development utilized Visual Studio (ver. 17.11.3) with C# (ver. 12) programming. Further details will be provided throughout this work. In addition, the materials, software, and libraries used in the development of this work are present in the Data Availability Statement. Both the development and experiments conducted with individuals were approved by the Ethical Committee for Human Research at the Federal University of Technology—Paraná (advisory number 7.218.395).

### 3.1. System Overview

**Figure 2:**
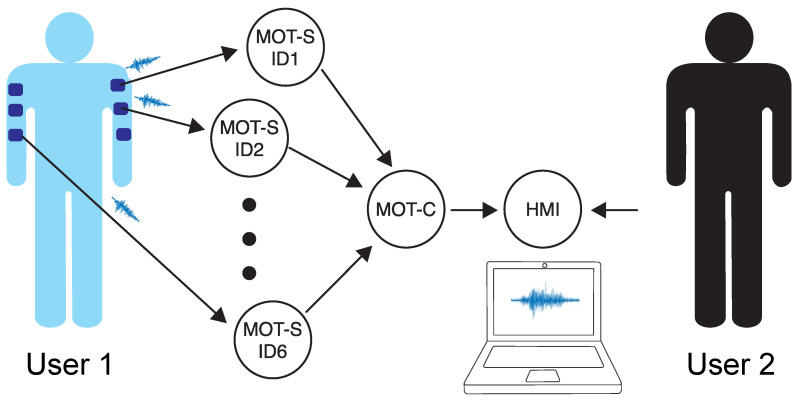
Diagram of elements in the developed system.

The MOT system is divided into three parts, as demonstrated in Figure 2. The first part is the sEMG sensors, with each abbreviated by MOT-S (MOT-Sensor). The MOT-Ss send the acquired signals for a central device (MOT-C—MOT-Central) via radio frequency (RF) communication. The process is unidirectional using the ESP-NOW (v1.0) protocol. The MOT-C is plugged into a personal computer by serial communication, and a human–machine interface (HMI) controls the operation, displays the signals in real time, and records them in text files.

Each MOT-S has its own number identifier (ID), which is predetermined from 1 to 6. The MOT-C receives messages from the MOT-Ss and then orders and organizes them to be sent to the HMI. The universal asynchronous receiver/transmitter (UART) performs the communication between the MOT-C and the HMI. The following sections describe the functionality of these elements.

### 3.2. Sensor Module: MOT-S

**Figure 3:**
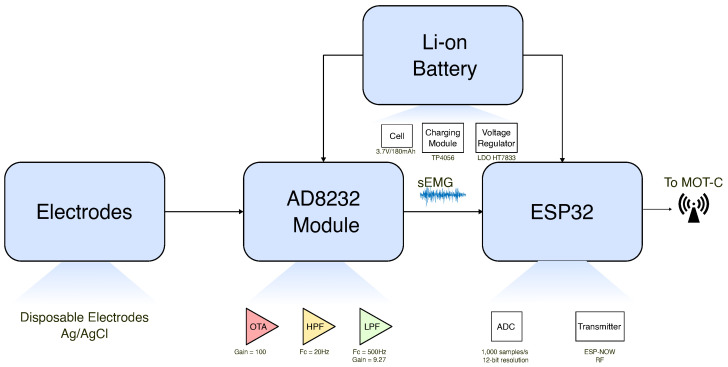
MOT-S diagram and its elements. Each MOT-S is made by an AD8232 module for sEMG acquisition, an electrode connection, and an ESP32 microcontroller. Each MOT-S is supplied with a Li-ion battery with a voltage regulator and a charging module.

The MOT-Ss acquire the sEMG signals; the hardware blocks are summarized in Figure 3. Signal capture and conditioning are handled by the AD8232 analog front-end device. Although conceived for single-lead ECG, the device has already been validated for sEMG acquisition [61,62]. Its high-pass (2nd order) and low-pass (2nd order) stages can be tuned simply by changing the external RC network [63]. Key specifications that motivated its selection include the following: supply range 2–3.5 V (compatible with battery-powered microcontrollers); quiescent current around 170 µA; and a common-mode rejection ratio of 80 dB, which is suitable to suppress main interferences [64].

The raw biopotential is first routed through an instrumentation amplifier that uses an indirect-current-feedback topology realized with two matched transconductance stages plus the on-chip DC-blocking network, delivering a fixed gain of 100 *V*/*V* while accommodating electrode offsets up to ±300 mV [65]. Posteriorly, there is a second-order high-pass filter with a 20-Hz cutoff frequency. This filter has unitary gain and is used to attenuate the movement artifacts, DC electrode level, and baseline noise. The next step is a second-order low-pass filter with a cutoff frequency of 500 Hz to delimit the frequency band of the sEMG signal and attenuate high-frequency noise. This filter was developed with a gain component set as 9.27. The “AD8232 Filter Design Tool" application was used to project these circuits. Moreover, AD8232 has a system that provides a DC offset for the signal, which is helpful for applications handling microcontrollers. In this application, the DC offset was half the supply voltage (1.65 V).

An ESP32-WROOM-32D module converts the analog AD8232 output to digital. Its on-chip SAR ADC offers a nominal 12-bit conversion, but with an effective resolution of about 10–11 bits; the converter is therefore configured for 83.3 kS/s (below the 200 kS/s limit quoted for RTC mode) to preserve linearity. The same module provides an integrated 2.4 GHz Wi-Fi/BLE radio and on-board antenna, meeting the project’s wireless communication and compactness requirements; the microcontroller has a specific protocol for radio communication named ESP-NOW [66]. It allows transmitting 250 bytes per message, an ACK for the transmitted message, and has lower latency than Wi-Fi and Bluetooth [67].

The MOT-S unit is powered by a 3.7 V, 180 mAh lithium battery (19 mm × 25 mm × 4 mm). The HT7833 voltage regulator maintains the voltage at 3.3 V. The IC TP4056 manages battery charging. Two LEDs were inserted to indicate the charging status: red for charging and green for fully charged. An electronic switch ensures safety during charging.

**Figure 4:**
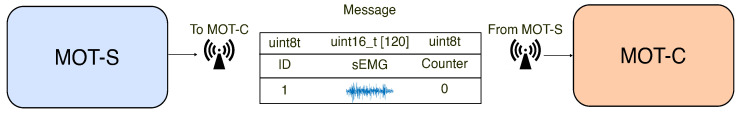
Structure of the messages sent from MOT-S for MOT-C.

Each MOT-S acquires the signal with a fixed sampling rate of 1 kS/s. The ESP-32 IC in the MOT-S uses a time interruption routine every 1 ms to follow the Nyquist criterion, as sEMG signals have a frequency band close to 500 Hz. MOT-S sends a message using ESP-NOW protocol with a channel Identifier (ID), the sEMG signal, and an incrementing counter, as demonstrated by Figure 4. Each message has a maximum size of 250 bytes [66,68]. The ID is used to order messages for HMI and attribute an identifier for the sEMG signals. These signals, acquired from ADC, are stored in a 120-position vector. The identifier is placed on the message, and it is used for the MOT-C to control the number of received messages. All these tasks were coded using the FreeRTOS (v10.5.1) library for parallel processing.

**Figure 5:**
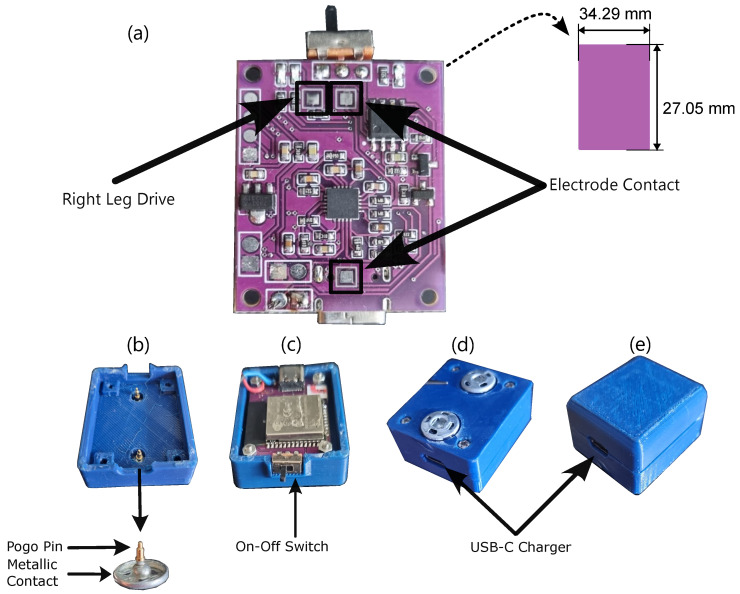
Detailing of the construction of MOT-S. The internal circuit is shown in (**a**). The contacts between the electrode and the circuit and the switch are presented in (**b**,**c**) in the upper and lower views, respectively, while (**d**,**e**) present the MOT-S’ case, highlighting the connection for charge for the upper and lower views.

Figure 5a presents the upper view of the MOT-S circuit board. The connections of the electrode contacts are highlighted. A second connection related to the right leg driver is shown. However, it is not used in this version of MOT-S, which could be used for future applications. These places are connected with pogo pins and metallic contacts, which interface MOT-S with disposable electrodes. They are presented in Figure 5b. These contacts put pressure between the electrodes and the PCB. A switch is responsible for turning on and off the device (Figure 5c). MOT-S complete view, with 3D printed Polylactic acid (PLA) case, is presented in Figure 5d,e, detailing the battery charger made by USB-C interface. MOT-S has 42.29 mm × 34.7 mm × 19.5 mm as final dimensions, weighing 21.94 g.

### 3.3. Central Module: MOT-C

**Figure 6:**
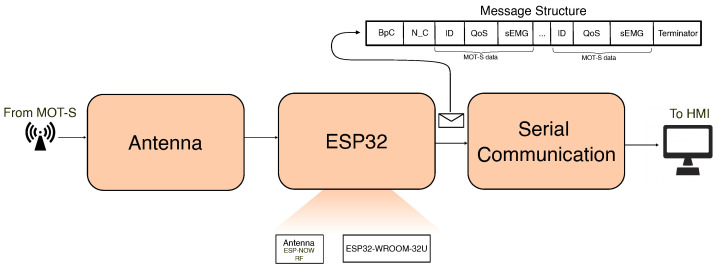
MOT-C diagram and its elements. The MOT-C comprises an ESP32, which receives the data from the MOT-S modules via an antenna and organizes the signals to be sent by HMI using serial communication. The message structure is presented in detail.

The MOT-C receives and manages the signals of all MOT-Ss and transmits them for the HMI. Internally, an ESP32-WROOM-32U microcontroller with an external antenna (8.75 mm) controls this module. A CP2102 integrated circuit commands the serial communication between the central and the HMI. An external voltage source from a USB-C connection (with 5 V) supplies this device, and a security system was developed to avoid damage from electrostatic discharges. Figure 6 presents the diagram of components for MOT-C.

The central unit software was developed using the FreeRTOS library to enable the parallel reception of messages and communication with the HMI. The central unit receives all messages for the MOT-S units. A logic in software was implemented to verify when the MOT-S was activated. It is divided into two events: the active channels send the ID in the message; when a channel stops sending messages for 1 s, it is considered deactivated. Messages are synchronized for all channels using a queue concept.

MOT-C communicates with the HMI by serial communication through a USB connector with a 921,600 baud rate. A protocol was defined to send data from MOT-C to HMI, ensuring that data were organized to be read by the interface to identify and prevent packet loss. It was implemented based on the principle of VoIP technology (Voice over Internet Protocol), utilizing a counter to organize the messages and verify the quality of received data. An example of this message is presented in Figure 6. The structure of the message includes several fields. The first message field is related to the Bytes per active Channel (BpC), which arranges the received data and verifies if there are multiple messages per channel transmitted from MOT-C to HMI. N_C field is the number of active channels; both BpC and N_C are the message headers. The following fields contain MOT-S data where the IDs of MOT-Ss are inserted, the quality of signal (QoS) of a message to guarantee their delivery, and sEMG data. The length of these fields corresponds to the number of active channels. Finally, a sequence is inserted to indicate the message’s end. QoS is evaluated as shown in Equation (1), where $cont_{\left[k\right]}$ and $cont_{\left[k-1\right]}$ are the “Counter” value present in the current and previous message sent by MOT-S.

(1) $$QoS=\sum_{k=1}^{N}\left(cont_{\left[k\right]}-cont_{\left[k-1\right]}-1\right)$$

**Figure 7:**
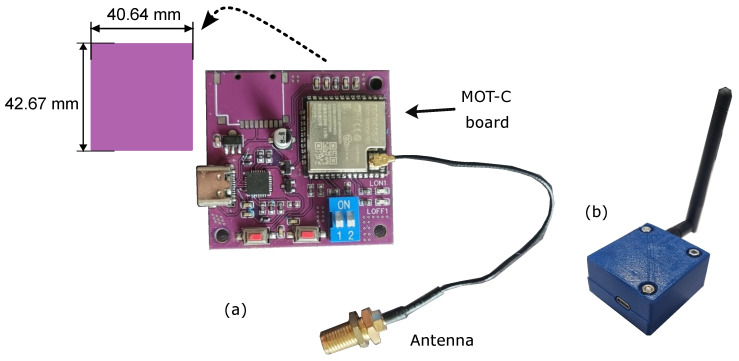
(**a**) Circuit Board of MOT-C. (**b**) MOT-C with the case.

MOT-C board is presented in Figure 7a, and the PLA case is presented in Figure 7b. The final dimensions of MOT-C are 46.64 mm × 48.67 mm × 23.2 mm, weighing 41.3 g (without the antenna). Pigtail cable aids the communication between MOT-C and MOT-S devices. The USB-C, viewed in Figure 7b, transmits the data for the IHM interface.

### 3.4. Interface

The HMI, developed using C#, enables online visualization and recording of sEMG signals. For this task, the interface has two main routines. The first is related to serial reading, which verifies the sequence of messages, since the data arrive asynchronously at the interface.

**Figure 8:**
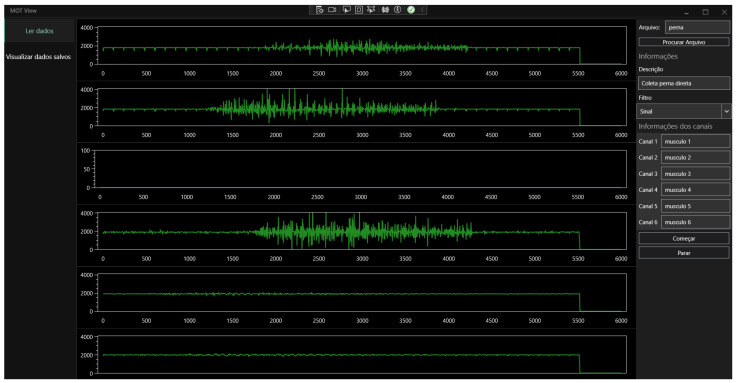
IHM developed in this work, with an acquisition example.

The open library OxyPlot (ver. 2.2.0) was used to display data. In addition, when data are displayed, HMI records the acquired signal into binary files. When the acquisition is over, the data are stored in a JSON file with the configuration parameters, such as signal description, channel name, QoS factor, sampling frequency, and analog-digital resolution. The example of one acquisition in the interface is presented in Figure 8. Channels 1, 2, 4, 5, and 6 are active, displaying the data simultaneously. When a channel is inactive, as on channel 3, no signal is shown in the interface. Independent of the number of channels, the MOT system did not interrupt the acquisition of other channels.

### 3.5. Experimental Methodology

**Figure 9:**
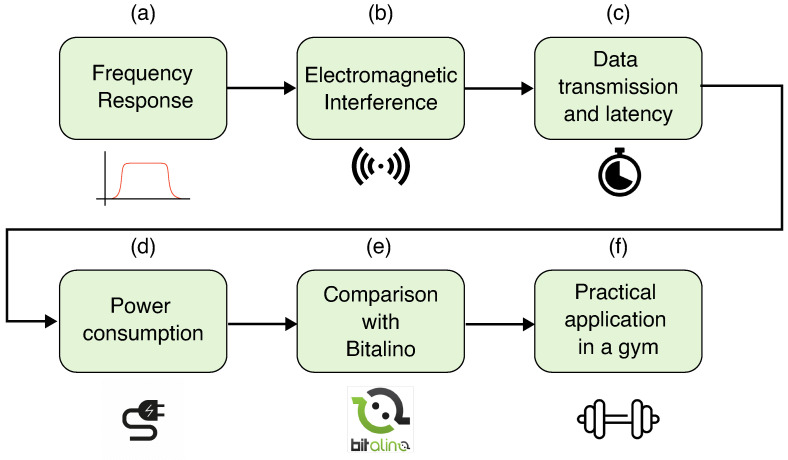
Experimental methodology steps followed to evaluate the MOT system. The frequency response (**a**) and electromagnetic interference (**b**) were examined from the MOT-S. Subsequently, the quality of data transmission of MOT-S for MOT-C was verified with the latency (**c**). The power consumption was measured and compared with a Bitalino device (**d**), along with a comparison with a signal acquired in the same condition by Bitalino and MOT (**e**). Finally, a practical application in a gym was conducted (**f**).

Some tests were conducted to verify whether the MOT system meets the proposed requirements. Two main types of experiments were proposed: one related to the MOT’s technical specification and one concerning a practical application. Figure 9 presents the steps developed in these tests. These experiments were conducted in two different places, a university laboratory and a gymnasium, to show potential applications of the device.

The first experiment evaluated the frequency response of the acquisition circuit, aiming to verify the filters’ cutoff frequencies and the amplifiers’ gain (Figure 9a). Sinusoidal signals with 3 mV amplitude and frequencies ranging from 1 Hz to 1 kHz, with 10 values per decade, were inserted into the input of MOT-S. A function generator AFG1022 (Tektronix) and an oscilloscope TDS2002B (Tektronix) were used to generate the sine waves and measure the output from the analog acquisition circuit of MOT-S. The magnitude of the Bode plot was extracted from the six MOT-S units, and for comparison, the output from the “AD8232 Filter Design Tool” software (ver 1.0.0.0) provided by Analog Devices was used.

The second experiment investigated the influence of electromagnetic interference in MOT-S (Figure 9b). Although batteries powered the MOT-S, without any cables between the electrodes and the acquisition circuit, this aimed to verify the influence of the main supply frequency (60 Hz) in the circuit. Six MOT-S units were placed on the biceps brachii, brachioradialis, and triceps brachii muscles of 5 volunteers at rest (without movements). Five seconds of signal were recorded, particularly capturing the electromagnetic interference. Then, the volunteers performed voluntary flexion of the biceps, and the sEMG signals were recorded during movement. Fast Fourier Transform (FFT) was used to obtain the power spectrum of the acquired signals, and an average of the channels was extracted to analyze the effects of electromagnetic interference.

The third experiment evaluated data transmission and QoS, as presented in Figure 9c. It assessed the impact of the operational distance and the number of active channels on transmission quality. QoS measures data transmission quality, defined by the number of package losses during communication (Equation (1). This metric was developed using a counter that registers the number of messages sent with each message sent to the MOT-C unit. The counter of the number of package losses is determined by comparing the counters of two subsequent messages. If the counter is not its successor, it indicates the difference between the lost packages. The sum of the packages during an acquisition was defined as QoS. To evaluate these metrics, the communication between the units of MOT-S and MOT-C was tested at distances from 0 m to 100 m, with a 10 m step, in an open field. The active channels varied from one to six in all conditions.

Data transmission latency measurement between the MOT-S and MOT-C units was conducted (Figure 9c). This process involved recording the timestamps when the MOT-C received the messages and calculating the interval between these received messages for each channel. To enhance the graphical representation in the HMI, the timestamps were adjusted by shifting the values in time so that the initial message of each channel was referenced at the start time (or time 0 s). The same protocol was applied to evaluate data transmission relative to distance. Thus, the obtained data were graphically represented, with time plotted on the x axis and the channels (IDs) on the y axis. This method facilitated the analysis of channel synchronization, the time difference between packages received from different channels during the acquisition, and the distribution of packages over time.

The next test concerning the technical specification of the MOT system focused on power consumption (Figure 9d). Moreover, a comparison with the Bitalino circuit was also conducted. This experiment used a power supply AFR FA3005-M to power the systems. The MOT was supplied with 3.7 V. The current consumption was measured for each circuit. For both circuits, only one channel was measured.

**Figure 10:**
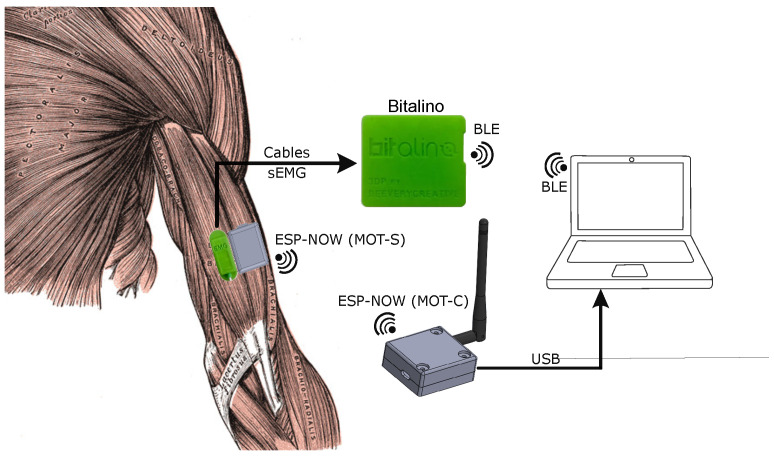
Schematic of simultaneous acquisition with the Bitalino and MOT devices.

Posteriorly, a comparison was made between the acquisition system and the Bitalino circuit to verify if they could provide a similar response for the same stimulus (Figure 9e). Figure 10 illustrates this process. Two pairs of electrodes were placed on the biceps brachii, with the MOT-S unit slightly to the left and the Bitalino to the right. A signal was recorded for five volunteers (5 males aged 20 to 26 years) who performed a lift with a 5 kg dumbbell. Bitalino signals were acquired using the software developed by PLUX Biosignals (Lisbon, Portugal), the OpenSignal (r)evolution (ver. 2.2.5), while the MOT interface was used to acquire the sEMG signals.

**Figure 11:**
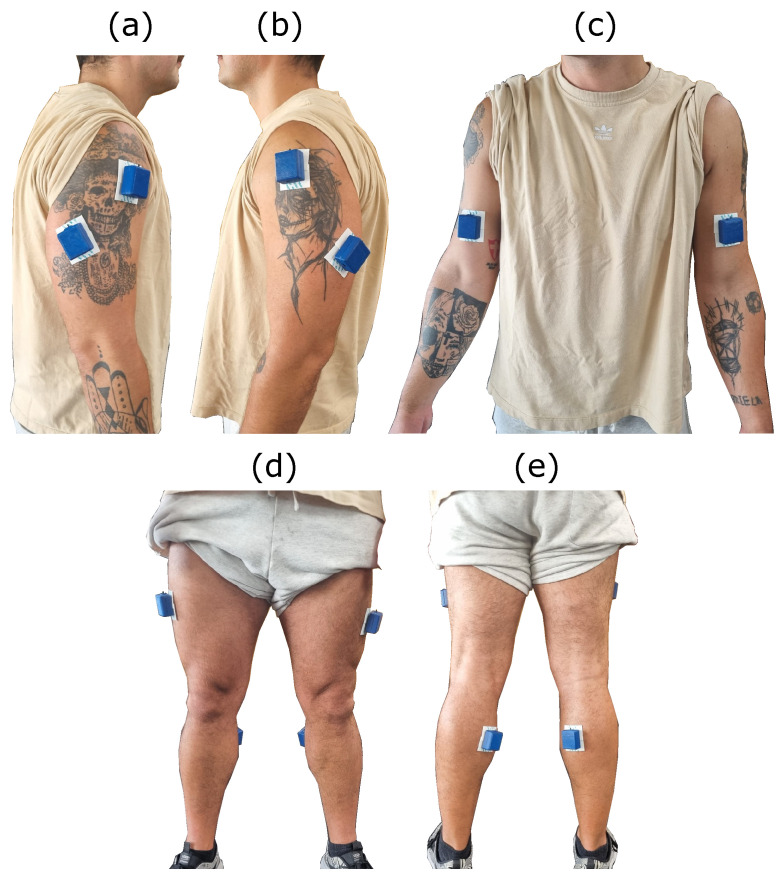
Positioning of electrodes in a volunteer: (**a**) right view and (**b**) left view of the deltoid and triceps, (**c**) frontal view with MOT-S placement on the biceps, (**d**) placement of the system on the vastus lateralis, and (**e**) placement on the medial gastrocnemius.

The last experiment was conducted in a gym to test the sensor under dynamic conditions similar to its practical application (Figure 9f). Six movements were chosen to evaluate the MOT platform, which are listed in Table 2. The electrode placement is shown in Figure 11. The sEMG data from 6 volunteers (3 males and 3 females) were acquired, and the data were analyzed during the movement execution. For these signals, analyses in the frequency domain spectrum, signal–noise ratio of channels, and the pattern of the movements were performed.

## 4. Results and Discussion

### 4.1. Frequency Response

**Figure 12:**
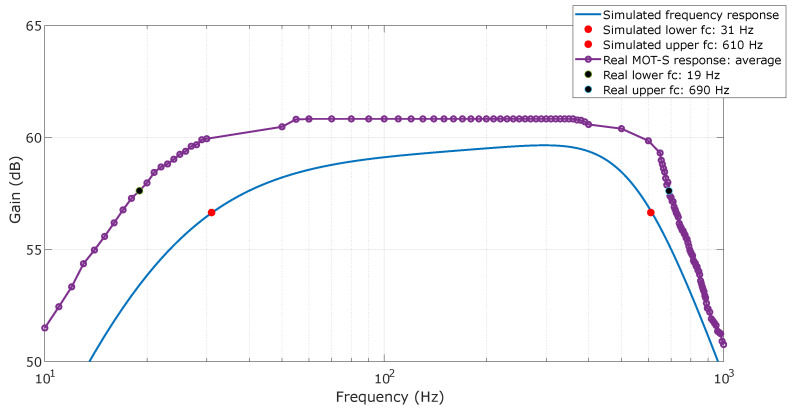
Frequency response (magnitude diagram) for MOT-S units. The response by the filter design application is shown with solid lines. The lines with dots are related to the MOT-S units’ response, representing the average of measurements. Red dots represent the simulated cutoff frequencies and black dots represent the average real cutoff frequencies.

Based on the frequency response tests, the cutoff frequencies were determined from the average measurements, as illustrated in Figure 12. Firstly, the continuous line shows the response obtained through simulation using the filter design and the projected components. The circuit’s gain for average frequencies was close to 60 dB (equivalent to amplification of 1000), with a slight increase up to 300 Hz. The cutoff frequencies were determined from the maximum gain: 31 Hz for low frequencies and 610 Hz for high frequencies.

Once the responses of MOT-S units were obtained, the maximum, average, and minimum values were extracted and the averages are shown on Figure 12. The gain of MOT-S units exceeded the project, which was not a problem during the sEMG acquisition, being near 60.7 dB for mean frequencies—in other words, the sEMG bandwidth. Also, this gain remained constant from 55 Hz to 360 Hz, which was different from the simulation. From the average of measures, 19 Hz was determined for the low-cutoff frequency and 690 Hz for the high-cutoff frequency. The high-pass cutoff frequency varied from 16 Hz to 23 Hz, while the low-pass cutoff frequency ranged from 655 Hz to 745 Hz. One can note that the maximum and minimum values exhibit some discontinuities. However, they are related to measurements for all six units, as the average measures the presented smoother transitions. The differences can be attributed to the tolerance of the components (capacitor (10%) and resistors (1%)) used to assemble the system. To elucidate this affirmation, if the value of the capacitors oscillates from minimum to maximum, the system’s cutoff frequency will be 1.11 or 0.9, respectively, times the expected frequency, since it is a second-order filter.

### 4.2. Electromagnetic Interference

**Figure 13:**
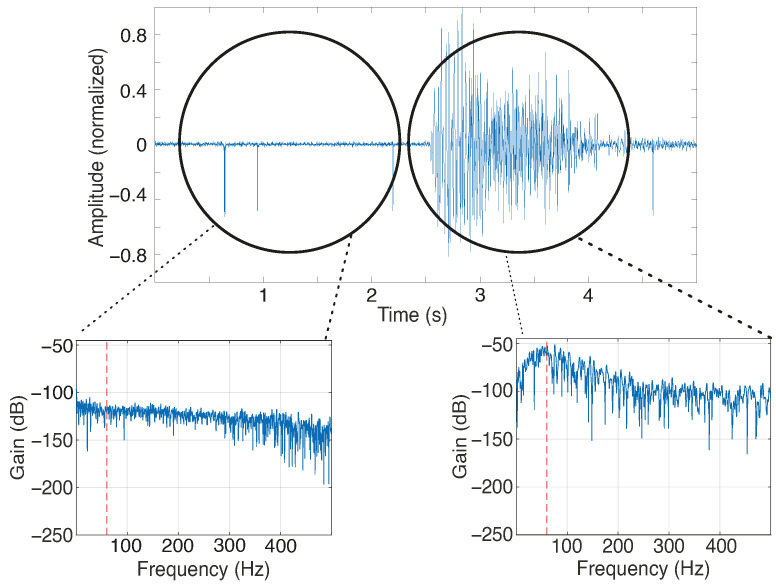
Example of the signal acquired in the test to verify the electromagnetic interference. The sEMG signal from the right bicep is presented, and the power spectrum in two instances (before and during the contraction) is presented. The red dashed line indicates the 60 Hz frequency.

The results obtained from the electromagnetic interference tests are presented in Figure 13. In Figure 13, an example of a signal from one channel obtained under two conditions (with and without muscle contraction) is presented, highlighting the frequency spectrum obtained under the two conditions. This signal has no processing, allowing for the visualization of whether a 60 Hz power noise interference is present along the sEMG signal.

From the power spectrum of Figure 13, one can see the frequency difference in the two scenarios. The 60 Hz signal has power near −110 dB under no-contraction conditions. During the acquisition, it is possible to note that the signals increased in sEMG bandwidth, including the 60 Hz frequency. However, as this frequency is within the sEMG band, one can note that the main power supply and other electromagnetic noise sources did not affect the acquisition to the point of invalidating a signal because of the noise level.

Thus, Figure 13 presents the difference between the rest and biceps flexion conditions obtained from the power spectrum for each channel for the 60 Hz frequency. One can verify that the difference between the conditions is higher in the biceps brachii and triceps brachii channels, which are more related to the chosen movement. The medians for these muscles are higher than 40 dB, representing a difference of more than 10,000 in power for the main supply frequency. The brachialis channels provided differences with medians close to 30 dB (or 1000 times in power), showing that even in muscles that are not fully contracted during an acquisition, MOT systems still acquire the sEMG signal with less influence of electromagnetic interference.

### 4.3. Data Transmission and Latency

**Figure 14:**
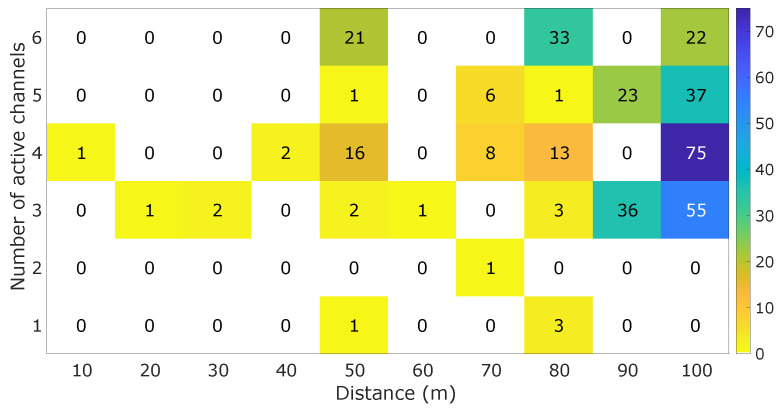
QoS obtained for different distances by different active channels.

Figure 14 presents the evaluation of the operational distance of the system as a function of the number of active channels. The “Number of active channels” variable is related to the determined quantity of active channels in the test, and it is not the ID of the MOT-S. One can note that the number of channels did not directly relate to the system’s maximum operational distance capacity. From the obtained values of the QoS, there is a proportional relationship between the distance and the number of lost packages. However, increasing the distance is not the only factor due to the lost packages, as observed in the case with six active channels, where 33 packages were lost in 80 m and 22 packages in 100 m, which could be related to external factors.

Upon analyzing Figure 14 in the two worst cases (both for 100 m and three and four channels), there was no visualized homogeneous distribution between the package losses. For three active sensors, one of the channels accumulated 48 of the 55 packages lost (85%), while for four active sensors, 54 of the 75 lost packages were concentrated in one channel, indicating more susceptibility of a fault sequence in the determined channels during the acquisition. Thus, one can note that for a distance of 100 m, the MOT did not present a significant package loss for one and two channels, which is considerable for acquisitions in this distance.

### 4.4. Power Consumption

Three different stages during MOT-S operation were observed. There was a current peak of 160 mA in the battery supply, followed by a stable consumption of 110 mA when disconnected from the MOT-C and close to 100 mA after connection of the MOT-C. Considering a battery with a capacity of 180 mAh, 1 hour and 48 min of autonomy was estimated for the system connected to the MOT-C. The charging process took 1 h and 19 min to complete the load. With the AFR FA3005-M source, it was observed that the current started at 145 mA, reducing to 5 mA. This process was assessed by the LEDs that indicated the charging status.

On the other hand, the device was compared against the Bitalino device in terms of power consumption. The Bitalino device consumption was measured at 10 µ A during one channel’s data transmission and acquisition. With a battery power of 170 mA, the time estimate of operation of the device is 17 h, assuming no power supply and consumption fluctuation. This difference is attributed to the communication protocol used, since the ESP-NOW protocol is responsible for a system consumption of 99.96 mA, and Bluetooth modules (such as HC-06) consume currents near 10 mA [69].

### 4.5. Comparison with Bitalino

**Figure 15:**
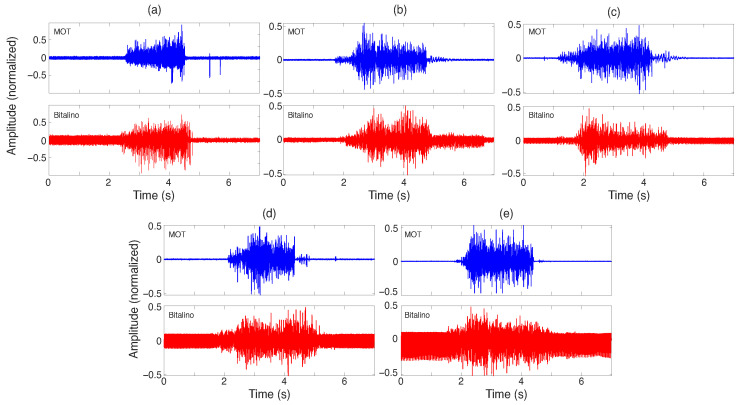
Signals acquired in the comparison with the Bitalino device for five volunteers, with (**a**–**e**) representing the volunteers 1 to 5, respectively.

**Figure 16:**
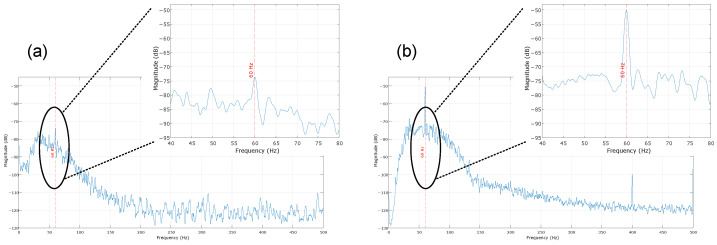
(**a**) Illustration of power spectrum from Figure 15a for MOT and (**b**) Bitalino response, detailing the power spectrum around 60 Hz.

Figure 15 presents the signals acquired simultaneously while comparing the MOT system with Bitalino, where the electrodes were placed on the right biceps brachii, and the volunteers performed a muscle contraction. The power spectrum for each signal was extracted. To exemplify this process, Figure 16 illustrates the power spectrum for the Figure 15a signals, highlighting a electromagnetic interference of 60 Hz frequency for both systems. Thus, Table 3 presents the magnitudes obtained for the power spectrum for each volunteer using the two systems to acquire the same muscle contraction.

From the previous figures, one can note that the MOT system presented a lower noise level than the Bitalino device (Table 3). For some signals, as shown in Figure 15a, mechanical shocks caused contact movement, which was passed to the signal. This could be attenuated with post-processing filters and by improving the interface of the contacts and electrodes. However, it did not modify the signal acquired by the system.

According to Figure 16, the MOT system presented a similar bandwidth for sEMG frequencies as the commercial device Bitalino. As the sEMG signals have energy in the band of 20 Hz up to 250 Hz, both systems showed higher magnitudes in this region, with the Bitalino device having a higher gain than the MOT device. The MOT device presented less variation at low frequencies than the Bitalino device, which did not impact the sEMG acquisition. Figure 15b details the influence of the primary supply frequency (60 Hz) on the two devices, with MOT showing a lower noise level. Additionally, one can note that the Bitalino signal has the presence of the main supply’s second harmonic (120 Hz) and a 400 Hz frequency component. The hypothesis to explain this effect is that it is due to the presence of cables in the Bitalino system during the transmission from the electrode to the processing device, although the latter transmits the data wirelessly [70].

### 4.6. Practical Application

**Figure 17:**
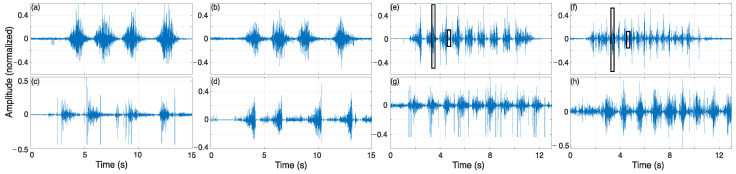
Examples of recordings from the practical application in the gym: (**a**) right deltoid and (**b**) left deltoid for dumbbell lateral raise; (**c**) right vastus lateralis and (**d**) left vastus lateralis for lunge; (**e**) right vastus lateralis and (**f**) right gastrocnemius for vertical jump; and (**g**) right vastus lateralis and (**h**) left vastus lateralis for squat.

For MOT practical application at a gym, Figure 17 presents some examples of raw signals obtained for the exercises mentioned in Table 2. The signals for the five volunteers are presented in the Data Availability Statement for future studies, with acquiring those data being one of the contributions of this paper: a dataset of signals for subjects performing gym movements for future works. One can note that the signals related to the superior limbs (Figure 17a,b) showed less interference from noise, which was due to movement artifacts and the main power supply.

However, some movements performed by the lower limbs (Figure 17c,e–g) showed some noise related to the movement development. It was not too perceptible in lunges, as visualized in Figure 17d. However, movements involving impact, such as vertical jumps, were transferred to the acquisition, as highlighted in Figure 17e,f. The impact of the user on the ground caused electrode shifting and high-frequency noise with short duration in the signal. However, it did not impact the acquired signal, as this noise had a higher amplitude level than the sEMG signal, and the designed filters attenuated it. Squat movements also showed noise related to the movement of muscles during the acquisition. This movement was performed without load (also known as an air squat), so it was more susceptible to the volunteer/user generating excessive muscle force during execution. When a movement was guided, such as with the use of dumbbells or bands, it was attenuated, as these movements have a more isometric behavior than free load. As the MOT system was able to identify these differences between the execution of movements, it can be used in dynamic movement applications, such as those developed in gyms or sports centers, with its wireless transmission being an alternative to facilitate the execution of movements by the user.

### 4.7. Comparison with Literature and Limitations

The MOT system demonstrated versatility for sEMG acquisition in these applications. In addition, it is easy to use and handle, with its main features presented in Table 1. Compared to the main features of the devices presented in Table 1, MOT operates in an RF range (2.45 GHz) similar to the works that did not employ cables for data transmission. Among the works with multiple channels, six have a similar number of channels to the MOT. The bandwidth of the developed systems (between 19 Hz and 690 Hz) is compatible with the analyzed works. Only three works have a cutoff frequency higher than the MOT system, which was not an obstacle to sEMG acquisition. Regarding sampling frequency, several works employed the same 1 kHz sampling frequency, but the resolution of the ADC was superior in nine works that reported this feature. Regarding acquisition system architecture, MOT is similar to [57], with the advantage of not depending on a physical connection (cables) between the acquisition system and the central system responsible for transmitting data to the personal computer.

Regarding the encapsulation and mass of the system, the work in [34] developed an acquisition device with dimensions of 90 mm × 50 mm × 25 mm and a mass of 102 g, being the system with the highest mass among those reported. On the other hand, the authors in [47] did not report the final mass of the equipment, which has dimensions of 57 mm × 38 mm, unlike the work of [39], where they only reported the mass of the developed equipment (23 g). The authors in [56] developed a device with dimensions of 39 mm × 32 mm × 17 mm and a mass of 24 g. The MOT system presented a lower mass, but close to that of the listed works (21.94 g).

The MOT presented a significant disadvantage compared to the listed works regarding power consumption. The work of [24] stands out, in which the system achieved an autonomy of up to two days for three active acquisition channels of sEMG, followed by [43], with an autonomy of 14 h. The MOT system did not present a superior performance in terms of operating time compared to the other works listed, being one of the limitations of this first version.

Among the tests performed by the other authors in the field and not only in the laboratory, the authors in [34] stand out. The authors performed running and stair climbing tests with the developed device and obtained sEMG signals from the rectus femoris and gastrocnemius muscles without the presence of noise caused by the impact. However, the researchers did not detail the method used to attach the equipment to the volunteer. The MOT system demonstrated robust performance in both controlled laboratory settings and diverse real-world environments. It successfully acquired sEMG signals over a 100 m open field and during practical applications within a gymnasium, emphasizing its adaptability and reliability across varied conditions.

## 5. Conclusions

This work presented the development of a multiple-channel sEMG system: MOT. It is compounded by six channels with wireless communication using ESP-NOW, with autonomy of 1 h and 12 min and low latency and with a distance of operation that can achieve 100 m for two channels and 70 m for all channels. In addition to the acquisition, a central system was developed to acquire the signals and an interface to present the acquisition and recording in real time for posterior analysis.

The MOT system was tested in terms of its behavior in a frequency band, electromagnetic interference, power consumption, latency, and data transmission, and it was compared against a commercial device (Bitalino) and in a practical application. In all tests, MOT met the proposed requirements. However, three main points require improvement and are recommended as future work. The first is related to the system’s energy efficiency, which presented a high current consumption, being approximately 92.5% less efficient than the system in comparison (Bitalino). This high consumption is attributed to the communication protocol we used (ESP-NOW). To solve this performance problem, it is necessary to change the communication protocol, for example, to BLE. The second point concerns the message organization architecture within the MOT-C. Queues were useful to prevent the microcontroller tasks from simultaneously accessing the same memory address and synchronizing the channels. Delays and accumulations of messages in the queues, due to retransmission or packet loss, caused a time lag in the information regarding the volunteers’ movements. The last point for improvement concerns the mechanical structure of the MOT-S. The collections performed in the gymnasium were efficient for the upper limbs, where there was no impact. However, after the feet impacted the floor for the lower limbs, the system recorded a high level of noise due to the shaking of the acquisition system. Despite the problems to be solved, the proposed system is still practicable in comparison with the other works, since it does not have cables connecting the electrode to the acquisition system. This reduces its susceptibility to power line noise or cable artifacts. Also, the power efficiency can be improved by changing the communication protocol or the system’s battery. However, besides its high consumption, the ESP-NOW allows for an acknowledgment from the transmitted message, enabling not only a packet loss quantification but also providing the user with when and how many times it happened, which is crucial in sEMG acquisition.

As possible solutions and future work, it is proposed to use other communication protocols (considering the maximum operating distance and energy consumption) to restructure the organization of messages received by the MOT-C (transferring responsibility for organizing messages to the HMI) and to reduce the size and mass of the MOT-S housing and hardware in order to minimize the impact on measurements involving high-intensity movements.

## Figures and Tables

**Table 2:** Details regarding the movements acquired in the practical test, the recorded muscles, and the number of repetitions. CH represents the muscle where the MOT-S unit was placed.

Movement	Action	Channels and Muscles	Repetitions
Squat	Execute squat with body weight and hip passing the knees	CH1: Right Vastus Lateralis CH2: Left Vastus Lateralis CH3: Right Gastrocnemius CH4: Left Gastrocnemius	3 series of 8 repetitions
Vertical Jump	Vertical jump with emphasis on impact	CH1: Right Vastus Lateralis CH2: Left Vastus Lateralis CH3: Right Gastrocnemius CH4: Left Gastrocnemius	3 series of 8 repetitions
Lunge	Alternate leg lunge with emphasis on muscle stability	CH1: Right Vastus Lateralis CH2: Left Vastus Lateralis CH3: Right Gastrocnemius CH4: Left Gastrocnemius	3 series of 8 repetitions
Dumbbell Lateral Raise	Raise the dumbbell with 20% of volunteer’s maximum load	CH1: Right Deltoid Medial CH2: Left Deltoid Medial CH3: Right Triceps Brachii CH4: Left Triceps Brachii	3 series of 4 repetitions
Triceps Push down with band	Push down with a band with focus on triceps movement	CH1: Right Deltoid Medial CH2: Left Deltoid Medial CH3: Right Triceps Brachii CH4: Left Triceps Brachii	3 series of 4 repetitions
Dumbbell Biceps Curl	Biceps curl with dumbbell at 20% of volunteer’s maximum load	CH1: Right Deltoid Medial CH2: Left Deltoid Medial CH3: Right Biceps Brachii CH4: Left Biceps Brachii	Maximum of voluntary repetitions

**Table 3:** Comparison of 60 Hz magnitudes (in dB) obtained from power spectrum for each signal of volunteers shown in Figure 15 between the Bitalino and MOT devices.

Volunteer	Magnitude (dB) MOT	Magnitude (dB) Bitalino
1	−73.60	−50.24
2	−78.23	−57.37
3	−92.64	−66.48
4	−82.49	−54.94
5	−75.75	−42.91

## Data Availability

Acquired dataset and supplementary files regarding the circuit can be found at this link: https://drive.google.com/drive/folders/11nO7BIljFxMjijcVaONMpNkiUYegHgWJ?usp=sharing (accessed on 6 June 2025).
